# Is it time for exercise-conditioned plasma to enter human trials?

**DOI:** 10.7150/ijbs.110586

**Published:** 2025-04-21

**Authors:** Patrick Henry Sebastian Sitjar, Jorming Goh

**Affiliations:** 1 Exercise Physiology & Biomarkers (EPB) laboratory, Yong Loo Lin School of Medicine, National University of Singapore (NUS), Singapore, Singapore.; 2 Healthy Longevity Translational Research Program (HLTRP), Yong Loo Lin School of Medicine, National University of Singapore (NUS), Singapore, Singapore.; 3 Centre for Healthy Longevity, National University Health System (NUHS), Singapore, Singapore.; 4 Division of Medical Oncology, National Cancer Centre Singapore (NCCS), Singapore, Singapore; 5 Department of Physiology, Yong Loo Lin School of Medicine, National University of Singapore, Singapore.

Chronic diseases, including cancer, cardiovascular diseases, metabolic diseases, and neurodegeneration, pose a significant burden to global health. While conventional pharmacological treatments have been first-line therapeutics for primary, secondary and tertiary prevention, innovative approaches are needed. In the past two decades since the sequencing of the human genome, there has been greater appreciation for the role of physical exercise in improving, maintaining or restoring homeostasis in the human body.

Skeletal muscle contraction during an acute bout of exercise elicits a complex array of molecular responses in multiple organ systems. Such molecular signals continue to persist, after the exercise and thus the long-term accumulation of such exercise sessions culminate in systemic adaptations that extend beyond the musculoskeletal system - remodeling of organ systems occur and improvement in healthspan^1^. Acute exercise mobilizes thousands of proteins and peptides, mRNA, extracellular vesicles (EVs) and non-coding RNA systemically, transporting them to distant sites, and exert modulatory effects on the organs, including brain, adipose tissue, liver etc^2^. Recent studies in pre-clinical mouse models reveal promising evidence that plasma obtained after exercise training directly improves physiological outcomes in non-exercised recipients. Transfused plasma from exercised rats improved neuronal viability, decreased cell atrophy and increased neurogenesis by three-fold in transgenic Alzheimer's Disease (AD) rat recipients^3^. Furthermore, exercised young (three-month old) murine plasma administered intravenously to old (18-month-old) mice resulted in increased proliferation of hippocampal neurons 4. Exciting work on plasmapheresis is being pioneered in the United States of America (USA) and Norway. In the former, young male donors provided 1 unit (~250mL) of fresh frozen plasma (FFP) to patients with AD in a once per week infusion, followed by a 6-week washout period and crossover with saline treatment. The primary endpoints were safety, tolerability and feasibility of the intervention - all of which were met at the conclusion of the trial^5^. In the latter, the ongoing study involved blood plasma obtained from young, healthy and well-trained (aerobically fit) individuals and transfused intravenously to older adults with Alzheimer's Disease at intervals of 3 months^6^.

Such recent investigations have given a glimpse of a novel translational application of exercise-induced adaptations for chronic disease management, particularly in oncology and neurology. In the next section, we elaborate on the molecular and cellular mechanisms that underlie the efficacy of exercise-induced plasma therapy, offering insights into its potential applications across diverse chronic disease contexts.

The release of molecular mediators known as exerkines during skeletal muscle contractions are partly responsible for the multi-organ health benefits derived from physical exercise. Broadly, exerkines can be classified into proteins and peptides, cytokines and chemokines, extracellular vesicles (EV), and metabolites.

Proteins and peptides released during exercise may modulate tumor growth and metastasis by regulating angiogenesis, immune surveillance, and tumor cell proliferation. Irisin, for instance, inhibits angiogenesis and suppresses tumor cell proliferation in various cancer models^7^. Glycosylphosphatidylinositol (GPI)-specific phospholipase D1 (Gpld1) was also identified as an exercise-induced, liver-derived enzyme (hepatokine) that confers cognitive function improvements to recipient aged mice via plasma administration from exercised donor mice^4^. Gpld1 upregulation was identified in active, elderly human subjects compared with sedentary controls, but it has not been elucidated if it offers cognitive benefits for humans through exercise^4^. Of note, *in vitro* culture of murine and human pancreatic islets treated with post-exercise training serum (10% v/v) reduced p16 and p21 cellular senescence markers, with further experiments indicating that, glucagon, behaving as an exerkine, could abate dysfunctional pancreatic metabolism patients with type 2 diabetes mellitus^8^. Recent advances in peptidomics have added novel candidates to the list of exerkines. For instance, the skeletal muscle-derived peptide, CCDC80tide, was released systemically after a single session of treadmill running in healthy young individuals^9^. This exerkine attenuated angiotensin II (Ang II)-induced cardiac hypertrophy and fibrosis in mice, primarily by modulating STAT3 phosphorylation.

Exercise-induced changes in cytokine and chemokine expression and release from different organs may exert anti-tumor effects by modulating immune surveillance and tumor microenvironment. IL-6, for instance, has been implicated in promoting anti-tumor immunity through its effects on T cell function and tumor-associated macrophages^10^. IL-15, with its immunostimulatory properties, enhances the activity of natural killer cells and cytotoxic T lymphocytes, thereby augmenting anti-tumor immune responses^11^.

EVs released during exercise may exert anti-tumor effects by delivering bioactive cargo, including miRNAs, proteins, and lipids, to recipient cells within the tumor microenvironment. Exercise-induced EVs have been shown to suppress tumor growth and metastasis by inhibiting angiogenesis, promoting immune surveillance, and inducing cancer cell apoptosis^12^. Moreover, EVs derived from exercised individuals can modulate cellular metabolism through an increase in concentration for proteins such as Suclg1, Sdha, Sdhb, Acly, Idh3b, and Dlat which are involved in mitochondrial biogenesis and β-oxidation^13^. Further, EVs are also understood to be highly heterogenous in nature making it difficult to isolate proteins or mRNA of interest, however, the encapsuled EV content are more resistant to degradation^13^.

During acute exercise, the production of metabolic byproducts may influence tumor metabolism and microenvironment, thereby affecting cancer cell proliferation, survival, and metastasis. β-hydroxybutyrate, a ketone body produced during fasting or low-carbohydrate states, has been implicated in suppressing cancer cell proliferation and inducing apoptosis through its effects on cellular metabolism and oxidative stress^14^.

Despite the therapeutic potential of exercise-conditioned plasma, such an approach requires extensive evaluation of possible complications. Sha *et al*, demonstrated the safety and tolerability of young FFP transfusion, but other unknown adverse reactions may mirror those experienced by patients in critical care units receiving transfusions such as febrile nonhemolytic transfusion reactions, anaphylactic reactions, transfusion-related acute lung injury (TRALI), transfusion-associated circulatory overload (TACO), etc 5, 15. Thorough medical screening of both donors and recipients especially for cardiac and renal function are necessary to mitigate such reactions 15. In addition, dosing regimens for exercise plasma transfusion are not known. The Alzheimer Symptom Amelioration study showed that weekly infusions of approximately 250 mL of plasma were well-tolerated by elderly Alzheimer's patients but further studies will be needed to uncover the optimal infusion protocol 5.

In summary, the putative molecular mechanisms that underlie the therapeutic effects of exercise-induced plasma transfusion therapy provide a foundation for their potential translational use in cancer, cardiovascular diseases, and neurodegenerative diseases. Furthermore, there is an opportunity to translate the benefits of exercise-induced plasma for bedridden or paralyzed patients who are unable or intolerant to exercise training. In conclusion, we believe it is time for early-phase clinical trials to test exercise-conditioned plasma for different chronic diseases. We envision a future where this therapy could enter mainstream medicine and call for collaborators to discuss multi-country clinical trials to explore this exciting space.

## Figures and Tables

**Figure 1:**
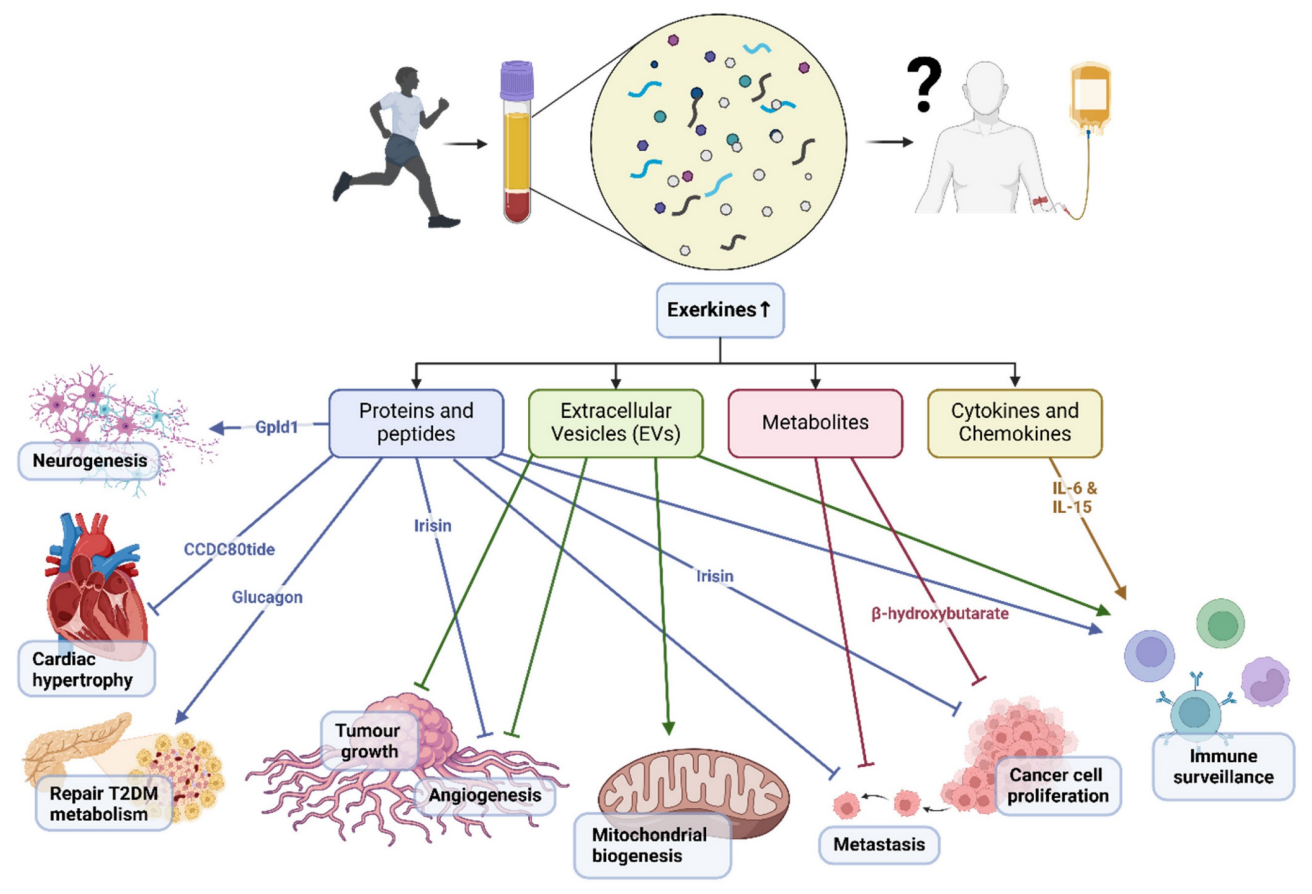
Graphical illustration showing a summary of the mechanisms of exercise-conditioned plasma on selected biological functions in health and disease.
